# Supplementary material for: An open-source framework for large-scale, flexible evaluation of biomedical text mining systems
Source: J Biomed Discov Collab. 2008 Jan 29;3:1. doi: 10.1186/1747-5333-3-1 (PMC2276192; doi:10.1186/1747-5333-3-1)
Supplement: Additional file 1 — Evaluation platform source code. The additional file contains the software evaluation framework discussed in this paper. The most current release can be downloaded from SourceForge.net [1]. See the accompanying README file for instructions on installation and use. Also included in the distribution is a collection of UIMA wrappers for some commonly used BioNLP tools and annotated corpora. [file 1747-5333-3-1-S1.zip › bionlp_uima_repository-1.0.1/docs/api/src-html/edu/uchsc/ccp/uima/ae/nlp/tokenizer/PennBioTokenizer_AE.html]

```
001    /*
002     *
003     * The Original Code is UCHSC UIMA.
004     *
005     * The Initial Developer of the Original Code is University of Colorado.  
006     * Copyright (C) 2005.  All Rights Reserved.
007     *
008     * UCHSC Utilities was developed by the Center for Computational Pharmacology
009     * (http://compbio.uchsc.edu) at the University of Colorado Health 
010     *  Sciences Center School of Medicine with support from the National 
011     *  Library of Medicine.  
012     *
013     *
014     * Contributor(s):
015     *   William A. Baumgartner <william.baumgartner@uchsc.edu> (Original Author)
016     *   
017     * Created: November 2005
018     * Dependencies:
019     * TODO:
020     *      - AnnotationSet should be set dynamically. Currently it is hard-coded.
021     * Changes:
022     *      01/10/06 - wab - adjusted to conform with the updated TextAnnotation class
023     *                     - it now uses the ClassMention, SlotMention, ComplexSlotMention framework
024     *      02/08/06 - wab - output is now TokenAnnotation, PhraseAnnotation, or ClauseAnnotation
025     *      02/23/06 - wab - output now included typed dependencies, tokens are now numbered
026     *      02/26/05 - wab - Token, Phrase, and ClauseAnnotations now have proper CCPClassMentions of their own
027     */
028    package edu.uchsc.ccp.uima.ae.nlp.tokenizer;
029    
030    import java.util.ArrayList;
031    import java.util.Iterator;
032    import java.util.List;
033    
034    import org.apache.uima.analysis_engine.ResultSpecification;
035    import org.apache.uima.analysis_engine.annotator.AnnotatorConfigurationException;
036    import org.apache.uima.analysis_engine.annotator.AnnotatorContext;
037    import org.apache.uima.analysis_engine.annotator.AnnotatorContextException;
038    import org.apache.uima.analysis_engine.annotator.AnnotatorInitializationException;
039    import org.apache.uima.analysis_engine.annotator.AnnotatorProcessException;
040    import org.apache.uima.analysis_engine.annotator.JTextAnnotator_ImplBase;
041    import org.apache.uima.cas.CASException;
042    import org.apache.uima.jcas.JCas;
043    
044    import edu.uchsc.ccp.uima.annotation.CCPDocumentInformation;
045    import edu.uchsc.ccp.uima.annotation.CCPTextAnnotation;
046    import edu.uchsc.ccp.uima.annotation.syntactic.CCPTokenAnnotation;
047    import edu.uchsc.ccp.uima.mention.CCPNonComplexSlotMention;
048    import edu.uchsc.ccp.uima.util.UIMA_Util;
049    import edu.uchsc.ccp.util.external.penn.PennBioTokenizer_Util;
050    import edu.uchsc.ccp.util.nlp.annotation.TextAnnotation;
051    import edu.uchsc.ccp.util.nlp.mention.ClassMentionTypes;
052    import edu.uchsc.ccp.util.nlp.mention.SlotMentionTypes;
053    
054    /**
055     * This class incorporates the StanfordParser into the UIMA analysis engine framework via the StanfordParserUtil.
056     * 
057     * 
058     * @author William A. Baumgartner, Jr.
059     * 
060     */
061    public class PennBioTokenizer_AE extends JTextAnnotator_ImplBase {
062    
063        private final boolean DEBUG = false;
064    
065        private PennBioTokenizer_Util pbt;
066    
067        /**
068         * The location of the serialized grammar file to be used by the Stanford Parser must be included in the descriptor file under
069         * "SerializedGrammarFile"
070         */
071        public static final String PARAM_TOKENIZER_MODEL_FILE = "pennTokenizerModelFile";
072    
073        /**
074         * Initializes the StanfordParser_AE by reading in the input parameters from the descriptor file and initializing a new
075         * StanfordParserUtil
076         */
077        public void initialize(AnnotatorContext ac) throws AnnotatorInitializationException, AnnotatorConfigurationException {
078            /* get input parameters from the descriptor file */
079            String tokenizerModelFile;
080            try {
081                tokenizerModelFile = (String) ac.getConfigParameterValue(PARAM_TOKENIZER_MODEL_FILE);
082            } catch (AnnotatorContextException ace) {
083                ace.printStackTrace();
084                tokenizerModelFile =  null;
085            }
086    
087            /* initialize a new StanfordParserUtil */
088            pbt = new PennBioTokenizer_Util(tokenizerModelFile);
089    
090            super.initialize(ac);
091        }
092    
093        /**
094         * This method processes the document text, and incorporates into the CAS TokenAnnotations, PhraseAnnotations, and ClauseAnnotations
095         * based on the output of the Stanford Parser
096         */
097        public void process(JCas jcas, ResultSpecification arg1) throws AnnotatorProcessException {
098            /* get document text */
099            String docText = jcas.getDocumentText();
100    
101            Iterator docInfoIter = jcas.getJFSIndexRepository().getAnnotationIndex(CCPDocumentInformation.type).iterator();
102            String docID = "-1";
103            if (docInfoIter.hasNext()) {
104                CCPDocumentInformation srcDocInfo = (CCPDocumentInformation) docInfoIter.next();
105                docID = srcDocInfo.getDocumentID();
106            }
107            
108            List<TextAnnotation> tokenAnnotations = pbt.getTokensFromString(docText, docID);
109            
110            /* add the TextAnnotations to the JCas */
111            UIMA_Util uimaUtil = new UIMA_Util();
112            uimaUtil.putTextAnnotationsIntoJCas(jcas, tokenAnnotations);
113    
114            
115            /* now, go through them, and add the more specific syntactic annotations, i.e. tokens, phrases, and clauses */
116            Iterator textAnnotIter = jcas.getJFSIndexRepository().getAnnotationIndex(CCPTextAnnotation.type).iterator();
117    
118            List<CCPTextAnnotation> syntacticAnnotationsToRemove = new ArrayList<CCPTextAnnotation>();
119            
120            while (textAnnotIter.hasNext()) {
121                CCPTextAnnotation ccpTA = (CCPTextAnnotation) textAnnotIter.next();
122    
123                String mentionName = ccpTA.getClassMention().getMentionName();
124    
125                if (DEBUG) {
126                    System.out.println(ccpTA.getClassMention().getMentionName() + "; " + ccpTA.getBegin() + " -- " + ccpTA.getEnd() + ": "
127                            + ccpTA.getCoveredText());
128                }
129    
130                if (mentionName.equals(ClassMentionTypes.TOKEN)) {
131                    if (DEBUG) {
132                        System.out.print("We are converting a SyntacticAnnotation into a TokenAnnotation");
133                    }
134                    /* if it's already a TokenAnnotation, then we don't have to do anything */
135                    if (!(ccpTA instanceof CCPTokenAnnotation)) {
136                        if (DEBUG) {
137                            System.out.println("... it was not a TokenAnnotation already.");
138                        }
139                        CCPTokenAnnotation tokenAnnotation = new CCPTokenAnnotation(jcas);
140                     try {
141                        UIMA_Util.swapAnnotationInfo(ccpTA, tokenAnnotation);
142                    } catch (CASException ce) {
143                        ce.printStackTrace();
144                    }
145    
146    //                    /* set the Annotator */
147    //                    tokenAnnotation.setAnnotator(ccpAnnotator);
148    //
149    //                    /* set the AnnotationSet(s) */
150    //                    tokenAnnotation.setAnnotationSets(ccpAnnotationSets);
151    
152                        /* set POS */
153                        CCPNonComplexSlotMention ccpSlotMention = (CCPNonComplexSlotMention) UIMA_Util.getSlotMentionByName(ccpTA,
154                                SlotMentionTypes.TOKEN_PARTOFSPEECH);
155                        if (ccpSlotMention != null) {
156                        tokenAnnotation.setPartOfSpeech(UIMA_Util.getFirstSlotValue(ccpSlotMention));
157                        }
158    
159                        /* set token number */
160                        ccpSlotMention = (CCPNonComplexSlotMention) UIMA_Util.getSlotMentionByName(ccpTA, SlotMentionTypes.TOKEN_NUMBER);
161                        if (ccpSlotMention != null) {
162                            tokenAnnotation.setTokenNumber(Integer.parseInt(UIMA_Util.getFirstSlotValue(ccpSlotMention)));
163                        }
164    
165                        
166                        /* remove the old annotation from the JCas, and add the new one */
167                        syntacticAnnotationsToRemove.add(ccpTA);
168                        tokenAnnotation.addToIndexes();
169    
170                    }
171    
172                } else {
173                    /* do nothing with anything other than token, phrase, or clause */
174                }
175    
176            }
177            
178            /* Removed the now unnecessary SyntacticAnnotations */
179            for (CCPTextAnnotation ccpTA : syntacticAnnotationsToRemove) {
180                ccpTA.removeFromIndexes();
181            }
182        }
183    }
```
